# The role of obesity and bariatric surgery-induced weight loss in breast cancer

**DOI:** 10.1007/s10555-022-10050-6

**Published:** 2022-07-23

**Authors:** Margaret S. Bohm, Laura M. Sipe, Madeline E. Pye, Matthew J. Davis, Joseph F. Pierre, Liza Makowski

**Affiliations:** 1 Department of Microbiology, Immunology, and Biochemistry, College of Medicine, The University of Tennessee Health Science Center, Memphis, TN 38163 USA; 2 Division of Hematology and Oncology, Department of Medicine, College of Medicine, The University of Tennessee Health Science Center, Memphis, TN 38163 USA; 3 Division of Bariatric Surgery, Department of Surgery, College of Medicine, The University of Tennessee Health Science Center, Memphis, TN 38163 USA; 4 Department of Nutritional Sciences, College of Agriculture and Life Science, The University of Wisconsin-Madison, Madison, WI 53706 USA; 5 Department of Pharmaceutical Sciences, College of Pharmacy, The University of Tennessee Health Science Center, Memphis, TN 38163 USA; 6 College of Medicine, UTHSC Center for Cancer Research, The University of Tennessee Health Science Center, Cancer Research Building Room 322, 19 S Manassas Street, Memphis, TN 38163 USA

**Keywords:** Immunotherapy, PD-L1, Obesity, High fat diet, Adiposity, Caloric restriction

## Abstract

Obesity is a complex metabolic condition considered a worldwide public health crisis, and a deeper mechanistic understanding of obesity-associated diseases is urgently needed. Obesity comorbidities include many associated cancers and are estimated to account for 20% of female cancer deaths in the USA. Breast cancer, in particular, is associated with obesity and is the focus of this review. The exact causal links between obesity and breast cancer remain unclear. Still, interactions have emerged between body mass index, tumor molecular subtype, genetic background, and environmental factors that strongly suggest obesity influences the risk and progression of certain breast cancers. Supportive preclinical research uses various diet-induced obesity models to demonstrate that weight loss, via dietary interventions or changes in energy expenditure, reduces the onset or progression of breast cancers. Ongoing and future studies are now aimed at elucidating the underpinning mechanisms behind weight-loss-driven observations to improve therapy and outcomes in patients with breast cancer and reduce risk. This review aims to summarize the rapidly emerging literature on obesity and weight loss strategies with a focused discussion of bariatric surgery in both clinical and preclinical studies detailing the complex interactions between metabolism, immune response, and immunotherapy in the setting of obesity and breast cancer.

## Introduction

Obesity is one of the most prevalent diseases in westernized societies, identified as a public health crisis both in the USA by the Surgeon General and worldwide by the World Health Organization [1, 2]. Based on body mass index (BMI; kg/m^2^), two-thirds of adults are considered obese (BMI > 30) or overweight (BMI 25–30), with one in three Americans identified as experiencing moderate (class 1: BMI 30 to 35 and class 2: BMI 35 to 40) to severe (class 3: BMI > 40) obesity [3, 4]. Surveys show increasing incidence, especially in women, and disproportionately high incidence in minorities [5–9]. Since a landmark publication by Calle et al. in 2003, obesity has increasingly been associated with specific types of cancer [10–12]. Currently, thirteen cancers are associated with obesity in terms of increased risk as defined by the American Institute for Cancer Research (AICR) [13], including postmenopausal breast cancer, colorectal cancer, endometrial/uterine cancer, esophageal adenocarcinoma, gall bladder cancer, gastric cancer, hepatocellular cancer, meningioma, multiple myeloma, ovarian cancer, pancreatic cancer, renal cancer, and thyroid cancer [11, 14]. Obesity is a significant, modifiable risk factor in nearly 20% of total US female cancer deaths and 30% of postmenopausal breast cancer cases [12, 15, 16]. Of particular interest for this review is obesity-linked breast cancer. With breast cancer being the most common malignancy and the second leading cause of cancer death in females [12, 17–26], understanding the links between obesity, adipose tissue, inflammation, immune response, and potential therapeutic interventions is critical to improving patient outcomes. This review will focus on the role of obesity in impacting breast cancer risk, prognoses, and survival, emphasizing the impacts of weight loss by bariatric surgery.

## Obesity and breast cancer

Obesity and breast cancer remain a complex conundrum that truly highlights the importance of including clinically relevant data in analyses such as tumor subtypes and menopausal status, ensuring adequate representation of diverse populations, predominantly minority and younger patients, and measuring several metrics to quantify obesity in complementary ways. For example, it is well established that obesity is a metabolically dysregulated state that increases the risk for postmenopausal breast cancer, primarily represented by the more prevalent luminal A subtype (ER-positive and/or PR-positive, HER2-negative [27]). In contrast, obesity in premenopausal women has been reported to be protective or have a null effect on breast cancer risk, typically when breast cancer is considered without accounting for subtypes [28–37]. However, many early studies were limited by lack of minority inclusion, few younger participants, a single measure of obesity, and estrogen receptor (ER) positivity determination only from immunohistochemistry (IHC) data. When studies include mainly white, older women who are more likely to present with luminal A and ER-positive subtypes, these studies may not be powered to detect the impacts of obesity on other breast cancer subtypes that strike younger or minority women. The decreased cost of sequencing has greatly increased the ability to quantify molecular subtypes.

Likewise, while BMI is convenient for extensive studies and retrospective analyses drawing from patient medical records with relatively little measurement error, BMI is fraught with limitations. Most notably, BMI lacks the ability to account for muscle mass compared to adiposity and fails to capture the compartmentalization of visceral versus central adiposity [38]. Other measures of obesity, including waist-to-hip ratio (WHR) and waist circumference, as well as more invasive measures such as DEXA scan or MRI, are complementary or improved metric options that are increasingly utilized in many studies. In addition, evaluation of obesity at cancer diagnosis may not be indicative of lifetime obesity exposure, hence some studies record obesity at a younger age along with current obesity status. Each strategy has pros and cons, influenced mainly by measurement error or cost. Likewise, the extent (classes 1–3) and duration of obesity are critical to consider. In sum, while BMI is convenient for population-based studies, including additional obesity measures adds to the ability to examine many important associations.

Once investigative teams began to oversample or enroll a disproportionately high percentage of minorities and younger women [27], include multiple breast cancer subtypes by additional IHC markers [39] or transcriptomic analysis, record pre- or postmenopausal status at the time of diagnosis, and use multiple measures of obesity, our understanding of obesity’s impact on risk became even more complex. In studies such as the Carolina Breast Cancer Study (CBCS) or consortia such as AMBER (African American Breast Cancer Epidemiology and Risk, which includes CBCS), obesity in premenopausal women was reported to be a risk factor for breast cancer, especially for triple-negative breast cancer (TNBC) [34, 40–43]. In contrast, several studies have not supported these findings, showing null results or moderately reduced risk ratios for obesity risk in premenopausal women. There is also evidence that overweight BMI reduces all-cause mortality of patients, promoting longer lifespans than normal-weight counterparts and reducing comorbidities [44], although controversy exists. Notably, a direct comparison of BMI to WHR suggests that WHR may better predict risk in African American women that is not detected by BMI alone, masking risk in many previous studies that only report BMI [40]. These findings exemplify the complexity of tumor subtypes, pre-or post-menopausal cancer, consideration of race and ethnicity, age when obese, duration of obesity, type of adiposity (visceral versus central), and use of BMI, WHR, or other quantification, which are all critical to consider in designing and evaluating studies of cancer risk. Furthermore, there is evidence that critically ill patients with obesity experience lower mortality rates [45]. This phenomenon is called the “obesity paradox.”

While the impact of obesity on cancer risk is complex, it is abundantly clear that obesity negatively impacts cancer outcomes and survival. In breast cancer patients, women with increased BMI have a higher risk of invasion [46, 47], distant metastases [48–50], tumor recurrence [51, 52], impaired delivery of systemic therapies [22, 53], and mortality [12, 54–60]. Thus, obesity represents a modifiable risk factor and is a target for cancer prevention measures and to improve cancer outcomes [61–63]. The exact molecular mechanisms linking obesity to breast cancer initiation (i.e., risk) or progression (i.e., outcomes) remain poorly characterized and are of great interest to the research community. As other recent reviews have examined the broad relationships between obesity, cancer, and the immune system [11, 64–66], this review will focus on the importance of modifying obesity-mediated breast cancer through various methods, focusing on weight loss induced by bariatric surgery. The field has much work ahead to integrate the many complex avenues of crosstalk in obesity and cancer. Still, there is great promise in identifying causal and targetable interactions in the obese tumor microenvironment.

## Weight loss and breast cancer

### Benefits of weight loss

Obesity is one of the few modifiable breast cancer risk factors [67], and weight loss has been proven to lower the risk of many cancers [68]. The risk reduction is most pronounced with intentional weight loss of ≥ 5% basal body weight among postmenopausal women [69, 70]. To date, most studies examining breast cancer risk are conducted with breast cancer survivors to measure recurrence rates as opposed to naïve patients being diagnosed with breast cancer for the first time. Weight loss improves overall prognosis [71] and reduces the risk of recurrence when combined with increased exercise [72–74], although not every study supports these conclusions [75]. Indeed, among breast cancer survivors, reduction in cancer recurrence resulting from weight loss varies by molecular subtype, though further analysis is needed to describe the differences [76]. Reduced risk of recurrence may be linked to weight loss-associated effects on circulating sex hormone levels [77]. In murine models, weight loss by caloric restriction has proven remarkably successful in reducing breast cancer progression, with intermittent caloric restriction proving more effective for lowering tumor incidence and size than chronic caloric restriction [78, 79]. Likewise, time-restricted feeding or time-restricted eating, also known as intermittent fasting, is successful for weight loss, tumor initiation inhibition, and tumor size reduction in both humans and mouse models [80]. Our group and others commonly use the relatively simple switch in dietary exposure from a high fat to a low fat diet to induce rapid and sustained weight loss [81, 82]. However, a diet switch approach includes both a change in dietary fat exposure and weight loss, which are difficult to disentangle when investigating mechanisms. Overall, findings support that weight loss by various weight loss methods such as dietary changes, caloric restriction, and increased exercise is beneficial in reducing both risk and recurrence, with the impact of bariatric surgery discussed in detail below.

### Lifestyle-induced weight loss and breast cancer

Lifestyle-induced weight loss relies on dietary changes, increased exercise, and similar adaptations to daily routines focused on lowering body adiposity and increasing physical activity. The Look AHEAD studies, for example, found that significant weight loss could be achieved and maintained over eight years. However, these results were most feasible when patients received a personalized lifestyle intervention plan and attended frequent check-ins with healthy lifestyle professionals [83]. However, a majority of studies suggest poor adherence to weight loss programs, with significant numbers of patients regaining lost weight or gaining weight over their starting point [84]. As introduced above, many studies on lifestyle-induced weight loss have been performed on breast cancer survivors because these cohorts typically show excellent study retention rates and adherence to lifestyle intervention plans [85]. Indeed, at-home intervention studies and supervised exercise programs have shown healthy changes in dietary behaviors and improved quality of life, along with weight loss and increased physical activity in long-term survivors [86, 87]. With regard to exercise alone, risk in murine models was first examined with supportive evidence showing that exercise reduces breast cancer incidence [88–90]. Indeed, voluntary exercise reduces tumor incidence and growth in mouse models as well as pre-operative breast cancer patients [91, 92].

Like obesity, exercise and dietary changes induce varied and complex benefits that can be linked to reduced cancer risk or progression from immunity to growth factors to adipokines to metabolism. For example, exercise upregulates anti-tumor immunity and downregulates immunosuppressive cells [92]. Others have reported that exercise intervention alone leads to reduced circulating levels of insulin, IGF-1, and leptin, improved natural killer (NK) cell cytotoxicity, or elevated adiponectin in patients [87, 93]. In patients, a healthy diet correlates to a reduction in circulatory estradiol and other sex hormones that promote oncogenesis [94], while fasting was associated with a decreased hemoglobin A1c and reduced risk of breast cancer recurrence [95].

In sum, published work supports the idea that healthy exercise habits and dietary choices are vital to reducing breast cancer risk or recurrence. This research area is expanding, with multiple randomized controlled clinical trials underway [74]. One such study, a large-scale, randomized phase III trial known as the Breast Cancer Weight Loss (BWEL, NCT02750826) trial, will examine the effects of weight loss due to lifestyle changes on disease-free survival among breast cancer patients [96]. Similar lifestyle changes have been shown to improve outcomes for other diseases associated with obesity as a modifiable risk factor, such as type 2 diabetes mellitus and associated cardiovascular disease [97]. Further study must be completed to draw any conclusions about reducing risk or improving survival with specific successful weight loss and lifestyle intervention approaches that are sustainable.

### Surgically induced weight loss and benefits

**Fig. 1:**
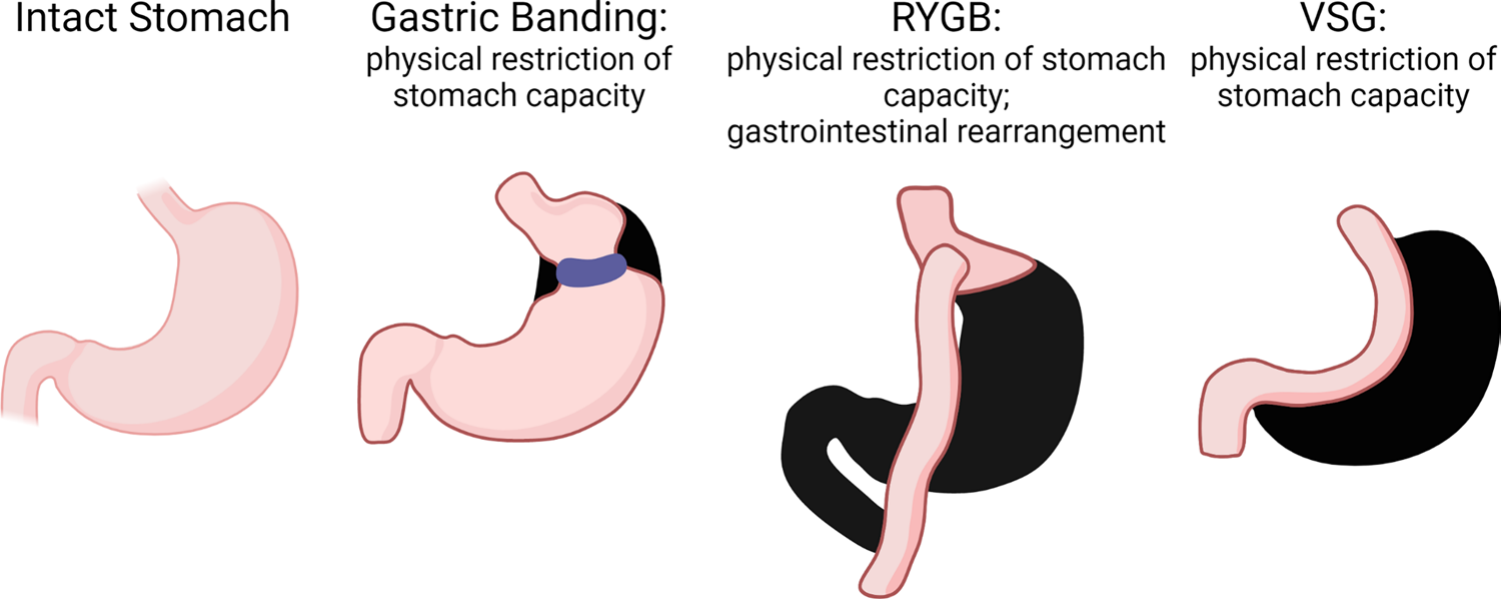
**Common bariatric surgery approaches**. Compared with the normal intact stomach, laparoscopic adjustable gastric banding (AGB) maintains the gastric cavity but restricts the fundus and cardia just below the esophagus to slow the rate of feeding and induce more rapid satiety. AGB was widely utilized but has fallen out of favor in the past 5 years due to adverse side effects, such as failure to sustain long-term weight loss, band slippage, and perforation. Roux-en-Y gastric bypass (RYGB) is both a restriction of stomach size leaving only a small pouch, as well as a dramatic rearrangement of the gut with impacts on gut peptides and gastric emptying. RYGB involves division of the upper gastric cavity from the lower, where the remaining duodenal pancreatic and hepatic secretions are diverted free of digesta and are anastomosed at a distal region of jejunum. Vertical sleeve gastrectomy (VSG) involves removal of the fundus and greater curvature portion of the stomach, leaving a sleeve that connects the esophagus to the duodenum. VSG is characterized by increased gastric emptying and hormone changes

The most effective method for long-term, sustained weight loss in adolescents and adults is bariatric surgery when combined with healthy lifestyle changes [98–102]. Surgical methods for weight loss were first introduced in the 1950s [103] yet did not gain wide prevalence in the USA or worldwide until the 1990s, when obesity rates and associated health conditions had risen to high enough levels to be considered an epidemic by the CDC and the WHO [1, 2, 104]. Historically, bariatric surgeries fall into two categories: restrictive or malabsorptive. Standard procedures performed today include adjustable gastric banding (AGB), vertical sleeve gastrectomy (VSG), and Roux-en-Y gastric bypass (RYGB) [105] (Fig. 1). AGB and VSG are restrictive gastric surgeries that primarily restrict or limit food intake. The resectional nature of VSG is also characterized by increased gastric emptying and hormone changes, leading to sustained, long-term weight loss. The RYGB, in contrast, is both a restriction of stomach size, leaving only a small pouch, as well as a dramatic rearrangement of the gut with impacts on gut peptides and gastric emptying. Gastric banding was widely utilized but has fallen out of favor in the past five years due to adverse side effects, such as failure to sustain long-term weight loss, band slippage, and perforation [106, 107]. The VSG, performed by removing the fundus and greater curvature of the stomach laparoscopically [108], is a technically more straightforward operation than the RYGB. In the USA, the VSG increased in prevalence from 11% to 70% of all bariatric surgeries performed from 2006 to 2015, while RYGB decreased proportionally [109]. While several studies show no significant difference in weight loss achieved and sustained by RYGB and VSG patients [110–112], others have shown that RYGB is more effective at supporting long-term weight loss than VSG [113]. Additionally, several studies have shown RYGB superior to VSG in comorbidity resolution, especially regarding diabetes [114]. However, the decreased level of invasiveness involved in VSG and its lower operative complexity have made it more appealing as an initial approach for many surgeons and patients.

Benefits of bariatric surgery include weight loss as well as many molecular changes that improve health. For instance, RYGB has five principle results, known as the BRAVE effects: bile flow alteration, reduction of gastric size, anatomical gut rearrangement with the altered flow of nutrients, vagal manipulation, and enteric gut hormone modulation [115]. Many of these effects are shared by the VSG procedure, except for anatomical gut rearrangement and possibly vagal manipulation. Both of these procedures are associated with an overall lack of hunger and improved gastric emptying [116]. These principles, primarily modulation of the gut microbiome, hormone signaling, and activity of various metabolic pathways, may be the true causes of both weight loss and many of the beneficial side effects of bariatric surgery, such as reducing comorbidities [116, 117]. Interestingly, in some patients, some benefits are rapidly induced after surgery, indicating they are not necessarily a result of the weight loss per se [118]. Additional mechanisms mediating the benefits of bariatric surgery include changes in endocrine signaling, microbiome, bile acid metabolism, and impacts on neuronal pathways [119–121].

Reduction in all-cause mortality after bariatric surgery ranges widely by study from 30 to 60% [122]. Patients eligible for bariatric surgery are characterized as those with BMI > 40 kg/m^2^ or 35–39.9 kg/m^2^ in combination with one or more obesity-associated comorbidities [3, 123]. Bariatric surgery results in sustained weight loss and decreased risk for multiple obesity-associated conditions [114, 124–128]. In the long term, bariatric surgery is also more cost-effective when compared to the financial burden of obesity-associated comorbidities [129], especially for women [130]. Despite these benefits, in 2018, bariatric surgery procedures were undertaken by less than 1% of the eligible population [3, 123]. There are several potential explanations for the low percentage of patients willing to undergo bariatric surgery. Bariatric surgery is an intensive procedure with long-term follow-up and lifestyle changes required. Patients must be healthy enough to experience such a surgery. Public distrust in the safety of such operations persists in the general population, partly because this surgery is intended for communities that are already more vulnerable to adverse complications of surgical procedures [131]. Other significant barriers include a lack of knowledge of the low risk yet high benefit of surgery by both patients and potential referring practitioners, a lack of insurance coverage, especially in public health plans, and the stigmatization of obesity, including the public perception of obesity as a lifestyle choice and not a disease. Currently, mortality rates are very low for all types of bariatric surgery, decreasing tenfold since the 1990s, and fall within accepted operative mortality rates at 0.3% for experienced hospitals [118, 123, 132]. Indeed, compared to the early 1990s, when open surgeries were the primary approach, laparoscopic surgery approaches now dominate bariatric surgery, which both improves outcomes and reduces recovery time [133]. Interestingly, benefits after bariatric surgery appear to be skewed towards women in the current research, as the cancer rates among men remained essentially unchanged [134]. The sex disparity in cancer risk could have biological underpinnings, but it should be noted that more women have undergone bariatric surgery and were available for analysis. Indeed, from 2002 to 2011, 80.7% of bariatric surgery patients in the USA were women [135]. Further study is necessary to determine the impact of bariatric surgery on male cancer risk and outcomes. Future studies are imminent, with the percentage of male patients receiving bariatric surgery increasing [135].

In sum, while bariatric surgeries are greatly increasing in prevalence, research is lagging to fully estimate the benefits of these approaches. As of 2022, 58.8% of studies had a follow-up time of just 1–2 years, which is a limitation to understanding the full impacts of these surgeries [136]. Thus, longer-term studies are needed to examine how and to what extent the underlying mechanisms of the benefits of bariatric surgery persist over many years.

### Bariatric surgery and breast cancer

Recent bariatric surgery studies have sparked great interest in the cancer field. Several retrospective studies reported reduced risk for some types of cancer as long-term benefits of bariatric surgery [134, 137, 138]. The decrease in all-cause cancer mortality varies up to 60% [122]. The SPLENDID study (Surgical Procedures and Long-term Effectiveness in Neoplastic Disease Incidence and Death) had a median follow-up of 6.1 years and examines a composite obesity-associated cancer score of 13 cancer types as the primary endpoint. SPLENDID results showed that bariatric surgery was associated with a 32% reduction in obesity-associated cancer and a 48% overall cancer-related mortality [139].

Regarding breast cancer specifically, bariatric surgery reduced breast cancer risk among postmenopausal women compared to non-surgical controls, with the most significant impact on risk reduction in ER-negative tumors, with a 64% decrease in risk [120, 134, 140]. Moderate declines in ER-positive [141] and HER2-positive breast cancer rates were reported [142]. An additional benefit to bariatric surgery is that subsequent cancers appear less aggressive or detected earlier. When breast cancer is diagnosed in patients after bariatric surgery, it is often diagnosed early as stage I with decreased prevalence of stage III or IV breast cancer [143]. Furthermore, bariatric surgery following remission from breast cancer resulted in sustained weight loss comparable to that of patients without a history of cancer. This may lengthen the disease-free survival time for breast cancer survivors [144]. In sum, bariatric surgery is promising for maintaining long-term benefits associated with weight loss with increasing evidence of reduced risk in some cancers, especially obesity-associated cancers. However, the benefits of bariatric surgery may not be so clear-cut. A potential complicating factor to the conclusion that bariatric surgery is protective against all obesity-associated cancers is evidence of an increased risk of colorectal cancer (CRC) after bariatric surgery [145]. Other studies have shown no increased risk of CRC associated with bariatric surgery [146]. This apparent discrepancy in risk of CRC likely reflects how the pathogenesis of cancer is heterogeneous and can be affected by different insults such as IBD, inherited germline mutations, and other factors. Further investigation is required to fully understand the benefits and risks of this life-changing surgery, especially concerning cancer risk of primary tumor development compared to recurrence. Table 1 highlights current clinical trials active or completed during this review.

**Table 1:** **Clinical trials from clinicaltrials.org**. The nine cancer-associated bariatric surgery human clinical trials are summarized by title, status, and conditions examined

NCT number	Title	Status	Conditions
NCT03946423	BAriaTric Surgery After Breast Cancer Treatment (BATS)	Not yet recruiting	Early-stage breast cancer|obesity
NCT04008563	B-FiERCE—Bariatric Surgery for Fertility-Sparing Treatment of Atypical Hyperplasia and Grade 1 Cancer of the Endometrium	Not yet recruiting	Endometrial cancer|atypical hyperplasia|bariatric surgery candidate
NCT04170335	Effects of Bariatric Surgery on Breast Density Improvement and Impact on Breast Cancer Risk in Severe Obese Patients	Recruiting	Breast cancer|morbid obesity|bariatric surgery candidate
NCT04839614	Concurrent Laparoscopic Hysterectomy and Weight Loss Surgery in Obese Patients with Endometrial Carcinoma or Endometrial Intraepithelial Neoplasia	Recruiting	Endometrial carcinoma|obesity|EIN|endometrial intraepithelial neoplasia|endometrial cancer stage I
NCT04284943	STARDOM- Surgical TreAtment for Obesity-Related Disease and Onco-Metabolic Surgery	Recruiting	Gastric cancer|diabetes mellitus, type 2
NCT01047735	TRIABETES- The TRIABETES—ARMMS-T2D Study: A Randomized Trial to Compare Surgical and Medical Treatments for Type 2 Diabetes	Active, not recruiting	Type 2 diabetes mellitus|obesity
NCT01922778	Screening for Endometrial Abnormalities in Overweight and Obese Women	Completed	Endometrial cancer
NCT02681120	Pilot Study of the Effect of Weight Loss on Breast Tissue and Blood Biomarkers in Women at Increased Risk for Breast Cancer	Completed	Breast neoplasms|obesity
NCT04345328	IMPORTUNE- Impact of Bariatric Surgery on the Gut Environment	Completed	Bariatric surgery candidate

## Modeling obesity in various mouse strains

Human cohorts offer insight into the effects of bariatric surgery and weight loss on cancer and other conditions. Still, current studies have been primarily retrospective, and this type of analysis is limited in the questions that can be answered. To be able to examine the mechanisms responsible for the benefits of weight loss, in vivo models are beneficial. Since the mid-1950s, researchers have been developing rodent models to mimic diet-induced obesity (DIO). Naturally occurring mutations in rodents and the development of transgenic mice complement DIO models, but single gene mutant mice are often not the most accurate representation of DIO observed in humans. Therefore, DIO and obesity-related conditions have been induced in murine models for decades by feeding the animals high fat diets; the effects are well-characterized [147, 148]. However, variability in diet, timing, and mouse genetics across studies often leads to inconsistent DIO findings [149].

To best conduct diet studies in pre-clinical models, it is vital to use defined, controlled diets that are matched on important dietary factors such as protein and micronutrient content. A major concern from a nutritional standpoint is that many researchers use chow to keep mice lean as the standard diet control as compared to DIO mice fed a defined high fat diet. This is adequate and understandably cheaper to maintain lean mice, but this approach is not ideal as a proper control. Micronutrients, fiber, and dietary components like phytoestrogens vary from lot to lot in chow, creating extreme variability in studies and introducing mediators that impact cancer outcomes [149]. Murine diets from commercial sources range from 45 to 60% kcal derived from fat, with 10% fat content typically used as the matched control for the high fat diets [149]. The main component in current commercial high fat diets is a high percentage of lard, or pig fat. However, lard is now consumed by Americans at much lower levels than were common decades ago [150, 151]. Still, lard-based diets remain the staple of pre-clinical DIO studies. Some dietary approaches modulate the fatty acid content [152]. Additional methods attempt to mimic the typical American or “Western Diet” more closely with a mixed diet of human foods, called the Cafeteria Diet [153–155], which varies widely from lab to lab. Others add cholesterol to a high fat diet to mimic Western Diet. Therefore, it is essential to note dietary interventions in manuscripts. Fortunately, investigators and journals pay increasing attention to diet details, exposures, and timing of diet intervention, which, taken together, will add to the clarity of our findings.

**Fig. 2:**
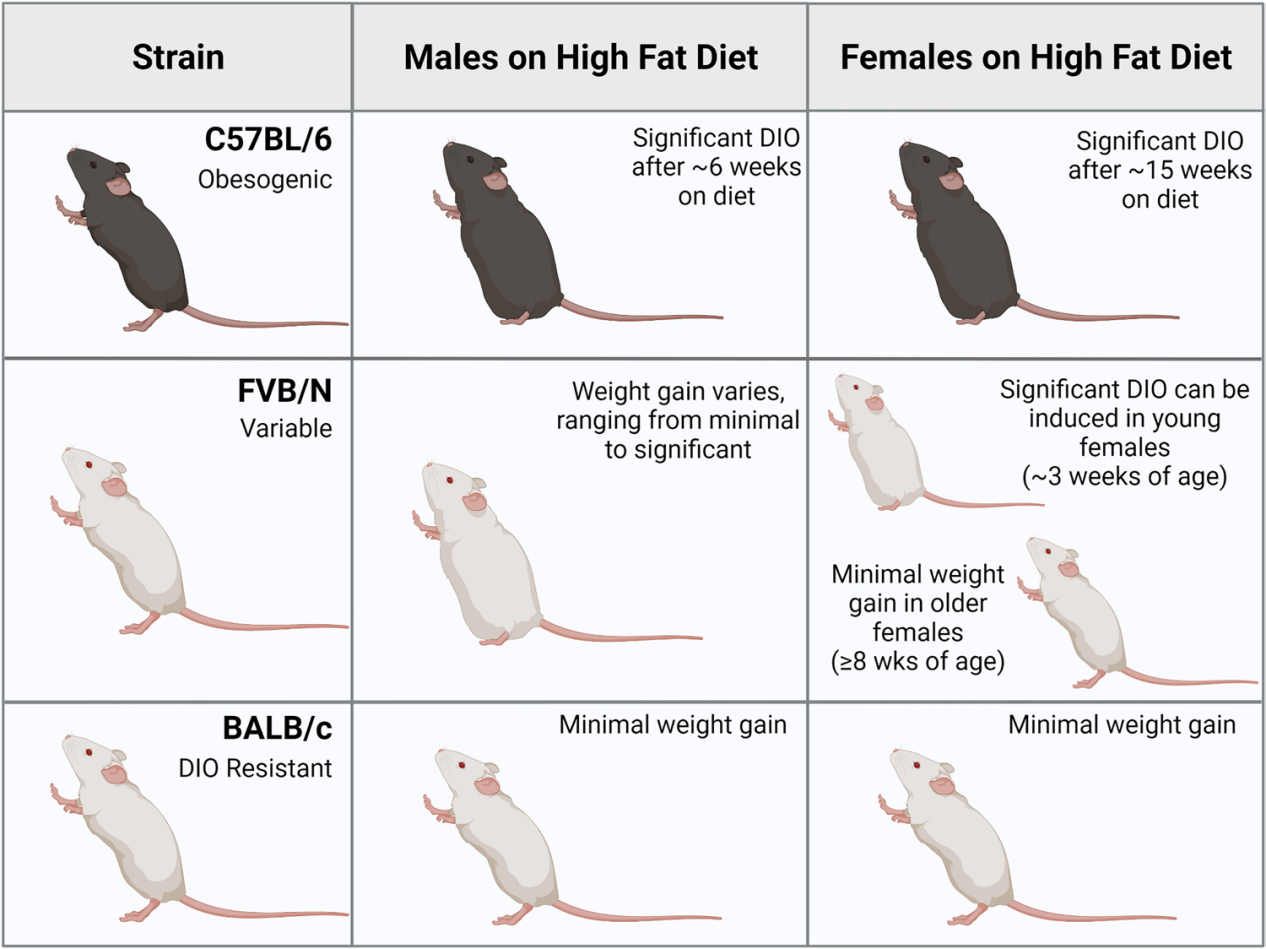
**Diet-induced obesity varies by strain and sex**. The susceptibility of murine models to diet-induced obesity (DIO) varies based on sex and background strain. C57BL/6 animals are highly obesogenic, where both sexes become obese, but males are more responsive to DIO compared to females with a more rapid and greater extent of adiposity observed. FVB/N animals exhibit highly variable weight gain based on the sex, age, and study in response to DIO. BALB/c animals are DIO-resistant regardless of sex or age

A critical factor in DIO studies is that mouse strains exhibit different extents of weight gain when exposed to high fat diets, just like individuals respond to various diets differently (Fig. 2). C57BL/6 mice are the classic strain used for many DIO studies because both sexes are readily obesogenic. C57BL/6 mice are highly susceptible to weight gain and tend to overeat on high fat diets [156]. Males tend to gain weight faster than females, with the male mice showing significant differences in weight gain between high and low fat diets after 6 weeks of diet, while females require 15 weeks to demonstrate such differences [157]. Notably, there can be variability in the C57BL/6 strain with some mice non-responsive to DIO weight gain. C57BL/6 variability in DIO is often attributed to differences in energy expenditure, ketogenic pathway regulation, or microbiomes [158]. Likewise, care should be taken to consider vendor and colony maintenance for consistency within studies. Mice from Jackson Labs, Inc. (C57BL/6 J) differ genetically from the NIH subline of C57BL/6 N (also sold at Jackson Labs), for example, with J or N in the strain name. The same is true for mice purchased from Taconic Inc. vs. Charles River Labs, Inc vs. Jackson, etc. [159], and the microbiome from these vendors also varies at baseline [160, 161]. Furthermore, mouse microbiomes may change for several reasons post-shipment, including transport from one building to another. These considerations are crucial for microbiome-dependent studies, as reviewed in Hugenholtz and de Vos [162]. Taken together, C57BL/6 mice are highly reliable obesogenic models but careful attention to study design should be implemented before and during DIO studies.

FVB/N mice are a strain commonly used for cancer studies but rarely used in DIO studies. In contrast to the C57BL/6 strain, the FVB/N strain shows significantly greater variability in responsiveness to DIO [150]. For example, substantial weight gain in FVB/N males has been published [163, 164]. In contrast, Boudina et al. showed resistance to high fat DIO and weight gain in FVB/N males [165]. Likewise, Devlin et al. reported females with no high fat diet-induced weight gain or increases in adiposity in FVB/N mice carried out to 20 weeks by body mass or body fat quantification [156]. In contrast, Zhu et al. published that a high fat diet following dimethylbenzanthracene (DMBA) exposure resulted in weight gain in FVB/N female mice carried out to 37 weeks of age [166]. Our group has extensive experience with a genetic model of cancer in the FVB/N strain. We reported moderate weight gain and increases in adiposity, with obesity-like detection of crown-like structures in adipose, and elevated obesity-associated endocrine factors, including leptin, using a transgenic breast cancer model in the FVB/N strain (C3(1)-T antigen, or C3(1)-Tag) [81, 82, 167–169]. We reported that obesity-like changes were reduced in C3(1)-Tag FVB/N mice by weight loss once obese mice were switched from a high fat diet to a low fat diet [81, 169]. Age and extent of exposure are critical to consider as well in DIO studies. In one study, young female mice introduced to a high fat diet at around 3 weeks of age gained significant amounts of weight, similar to that of age-matched C57BL/6 mice, when compared to low fat diet controls; however, when a high fat diet was introduced at 8 weeks of age, the response to diet was much less dramatic [170]. Mild weight gain was detected in older FVB/N female mice exposed to a high fat diet [171], but the difference in weight gain between low and high fat diet-fed mice was inconsequential compared to that observed in C57BL/6 female mice at the same age. Thus, FVB/N mice remain relatively controversial, with results across the spectrum of DIO. Depending on the study, FVB/N are generally perceived to be either resistant to DIO, moderately susceptible to becoming overweight but perhaps not frank obesity, or an adequate model for DIO. In sum, the model must be tested in each lab’s specific animal facility with diet details and timing of dietary exposure adequately controlled for adequate determination of DIO in FVB/N mice.

Interestingly, a third strain commonly used in cancer studies is BALB/c. It is well-established that BALB/c mice display minimal to no weight gain on a high fat diet compared to C57BL/6 and FVB/N strains. Indeed, the strain is often termed “obesity-resistant” [172]. In male BALB/c mice, high fat diet induced minimal weight gain but to a far lesser degree than that measured in C57BL/6 mice [173]. However, increases in colon cancer progression and metastases were shown in BALB/c males consuming a high fat diet, suggesting impacts of diet alone in the absence of DIO [174]. High fat diet induced changes in metabolite profiles despite the lack of significant weight gain [175]. In female BALB/c mice, a high fat diet increased tumor progression and metastases following injection with 4T1 breast cancer cells [176, 177], while others have shown that HFD (with no weight gain) did not increase 4T1 progression [178]. Taken together, high fat diet in the DIO-resistant BALB/c needs further study. Like other strains, timing of diet intervention is critical. The introduction of a high fat diet during pubertal mammary gland development, which is 3 to 6 weeks of age, led to weight gain and changes to the mammary duct development in C57BL/6 but not BALB/C female mice of the same age [179]. However, a high fat diet during this pubertal period in BALB/c females reduced carcinogen-induced tumor latency. High fat diet elevated inflammatory factors, supporting the importance of diet exposure in the absence of obesity and understanding critical windows of exposure susceptibility [180]. In sum, the BALB/c model is excellent for studying diet-induced cancer risk or progression but fails to induce frank DIO. Thus, if the intention is to research obesity per se, the BALB/c strain is a poor model, especially when investigating weight loss impacts after dietary interventions or bariatric surgery.

Last, examining patient-derived cell lines or xenografts (PDXs) in the obese setting introduces another layer of complexity. Immunocompromised murine models are necessary to avoid rejection of human cell lines or patient-derived samples. However, there are immunocompromised mouse strains, such as severe combined immunodeficiency (SCID) mice [181], that may be susceptible to DIO, including obesogenic (C57BL/6 J or C3H/HeJ), or DIO resistant (BALB/cBy), or NOD/ShiLtSz background, which is variable in response to HFD depending on background [182, 183]. NSG mice are from Jackson Labs on the background NOD/ShiLtJ and are commonly used in PDX studies, but their response to HFD appears variable. These strains are just some of the available options for PDX studies. The genetic background of the strain used must be noted and DIO responsiveness carefully considered based on the research question.

Overall, numerous studies use the three primary mouse strains discussed here, C57BL/6, FVB/N, and BALB/c, as models of DIO and diet-induced weight gain or resistance. There are multiple preclinical models of DIO to examine cancer risk and progression using carcinogen initiation, transgenic oncogene expression, syngeneic cell implantation, and more, which will be highly informative to the field. Primary study endpoints include obesity per se, specific dietary impacts, and the extent of weight gain or adiposity impacting cancer outcomes. The intention of the study design should inform model selection.

## Modeling bariatric surgery in preclinical setting

DIO and dietary interventions to induce weight loss, such as changing diet, timed feeding, fasting, and caloric restriction, are increasingly common in cancer outcomes studies. However, examining bariatric surgery after DIO is a little studied area that holds great interest based on the epidemiological findings about bariatric surgery and cancer risk discussed above. One major limiting factor is the skill set necessary to perform these complex, expensive, and time-consuming surgeries. Despite the technical challenges of mouse survival surgery, multiple groups have employed surgical models throughout the last decade to examine a wide range of obesity-associated conditions ranging from diabetes to cardiovascular disease and even gut-brain crosstalk. This field is growing immensely with over 170 publications on PubMed in the last year alone using rodent models of bariatric surgery. A selected subset of manuscripts published since 2010 are summarized in Table 2.

**Table 2:** **Rodent models of bariatric surgery highlighted in this review**. The results of 20 preclinical studies from 2010 to 2021 are summarized based on the rodent model, sex included, type of bariatric intervention, primary outcomes, and study results [184–187, 189–194, 196–199, 202, 203, 205, 206, 312, 313]

Study	Model	Sex	Surgical techniques	Primary outcomes	Study results
Stepfather et al. 2010	Long-Evans rats	Male, female	VSG	Weight loss	Weight loss is not sustained over time
Chambers et al. 2011	Long-Evans rats	Male	RGB, VSG	Weight loss and glucose regulation	Both methods had similar weight loss and improvement in glucose regulation
Yin et al. 2011	C57BL/6, FVB/N mice	Not specified	Gastric banding, VSG, RYGB, modified RYGB, biliopancreatic diversion	Comparison of weight loss induced by multiple bariatric surgery procedures	RYGB and biliopancreatic diversion are the most effective but also have the most side effects
Asian et al. 2012	Long-Evans rats	Female	RYGB	Effects of estradiol on weight loss and efficacy of satiation signaling	Estradiol increases weight loss and satiation signaling
Yin et al. 2012	C57BL/6 mice	Not specified	Duodenal-jejunal bypass, gastric banding, VSG, modified RYGB, biliopancreatic diversion	Comparison of weight loss induced by multiple bariatric surgery procedures	Duodenal-jejunal bypass is more effective than VSG, but more side effects
Brinckerhoff et al. 2013	Sprague Dawley rats	Female	VSG	Weight loss, adiponectin, and leptin levels	VSG results in weight loss and improvement of adiponectin and leptin levels
Grayson et al. 2013	Long-Evans rats	Female	VSG	Effects on the female reproductive cycle and their offspring	VSG improved the reproductive process, but offspring on HFD were predisposed to glucose intolerance and obesity
Liou et al. 2013	C57BL/6 J mice	Male	RYGB	Adiposity and gut microbiome composition	Shifts in microbiome composition contribute to decreased adiposity
Saedi et al. 2013	Long-Evans and Goto-Kakizaki rats	Male	RYGB	Weight loss, glucose metabolism	RYGB in rodents improves glucose metabolism as seen in diabetic humans
Ryan et al. 2014	C57BL/6 J FXR KO and W.T. mice	Male	VSG	Effects of FXR signaling on feeding, glucose tolerance, and gut microbiome composition	Bile acid signaling plays a critical role in modulating the results of VSG surgery
Hao et al. 2016	C57BL/6 J mice	Male	RYGB	Body weight, composition, food intake, and energy expenditure	Weight loss is achieved through increased energy expenditure, not decreased food intake
Frikke-Schmidt et al. 2017	C57BL/6 J mice	Male	VSG	Adipocyte tissue leukocyte profile	VSG causes changes in adipose immune populations, regardless of weight loss
Grayson et al. 2017	Long-Evans rats	Male, female	VSG	Sex differences in fat loss, hepatic gene expression, and cholesterol metabolism	Males see a reduction in hepatic lipids while females see an increase in the hepatic inflammatory pathway; VLDL was decreased in females alone
Hao et al. 2017	C57BL/6 J mice	Male	RYGB, VSG	RYGB vs. VSG in weight loss, food intake, energy expenditure, and glycemic control	RYGB is more effective in inducing weight loss and improving glycemic control than VSG
Spann et al. 2018	Long-Evans rats	Female	VSG	Effects of VSG on DIO dams	VSG reduces T cell populations, increases IL-1β, and increases placental permeability contributing to fetal demise
Spann et al. 2019	Long-Evans rats	Female	VSG	Effects on the immune system of offspring	The immune system is compromised early in life but rebounds after weaning
Ye et al. 2020	C57BL/6 J mice	Male	RGB, VSG	Changes in energy balance and browning of visceral fat	RYGB specifically increases resting metabolic rate and fat browning compared to VSG
Chaudhari et al. 2021 A	C57BL/6 J mice	Male	VSG	Changes in bile acid levels, glucose metabolism	VSG causes an increase of a TGR5 agonist, indicating the role of bile acid signaling
Chaudhari et al. 2021 B	C57BL/6 J mice	Male	VSG	Changes in the gut microbiome, bile acid levels, changes in bile acid signaling	VSG induces microbiome shifts that increase bile acid signaling, and this can be replicated in germ-free animals following FMT from VSG animals
Stevenson et al. 2021	C57BL/6 mice	Male	RYGB, VSG	Post-op effects of HFD on adipose tissue composition, weight alterations, and metabolic dysregulation	RYGB causes lasting improvement in these outcomes, while VSG is comparable to sham control

Both rat and mouse models have been used to examine the effects of surgical weight loss following DIO. In 2010, Stefater et al. described an early method for reviewing the weight loss induced by bariatric surgery in both male and female rats. They showed that in the long term, weight rebounded due to changes in eating habits resulting in a lack of changes in overall calorie intake [184]. The same group then compared the efficacy of RYGB against VSG in Long-Evans rats; males were fed a 41% high fat, butter oil-based diet and subjected to RYGB, VSG, or accompanying sham procedures to show that both RYGB and VSG resulted in similar patterns of weight loss and glucose regulation [185]. Later work by Saeidi et al. demonstrated that the improvements to glucose metabolism observed in humans with diabetes are recapitulated in rats following RYGB [186].

In 2011, Yin et al. developed five types of bariatric surgery in C57BL/6 and FVB/N mice to treat DIO resulting from a 60% high fat diet [187]. This study was likely conducted in males, though not specified. The surgical models included gastric banding, sleeve gastrectomy, RYGB, a modified RYGB better adapted to mouse survival, and biliopancreatic diversion [187]. Biliopancreatic diversion combines sleeve gastrectomy with an intestinal bypass in a highly invasive method previously reserved for individuals with BMI > 50 kg/m^2^ [188]. Recent innovations to decrease complexity make this procedure more attractive to patients and surgeons. Although biliopancreatic diversion and RYGB resulted in the most pronounced weight loss, a higher risk of side effects, such as anemia, was observed in the mice undergoing these procedures, which mirrors the results in patients. Further development in 2012 led to the implementation of the duodenal-jejunal bypass procedure in mice, which involves the removal of the duodenum from the gastrointestinal tract performed on some patients [189]. Like RYGB and biliopancreatic diversion, the duodenal-jejunal bypass procedure also showed worsened side effects for the mice than the sleeve gastrectomy. Despite the concerns for anemia, multiple studies have successfully used the RYGB surgical approach in preclinical models to examine its effects on estradiol metabolism, body composition, and the nervous system [190–192]. More recent studies have used C57BL/6 mice to compare the efficacy of RYGB versus VSG as it relates to sustained weight loss and glycemic control [193–195], with RYGB producing better results. However, the VSG was effective with significant weight loss with minimal side effects, and it is often used today in preclinical studies. Interestingly, VSG can also alter the immune systems of male C57BL/6 mice in a weight-independent manner [196], and many of the surgery results are influenced by bile acid signaling [197–200].

A significant factor to consider in interpreting studies, especially about cancers primarily or solely impacting women, such as breast, ovarian, or endometrial cancer, is that most published DIO bariatric surgery studies are completed in male rodents. There are several pros to using male rodents. First, male rodents gain much more weight on a high fat diet and gain that weight faster than females, as noted above. Second, large commercial labs like Jackson Labs, Inc. sell obese males on a high fat diet which makes completing DIO studies much more rapid and feasible. Unfortunately, Jackson Labs does not provide the same DIO service for female mice. This means that each study on female mice must be completed in house over many months to induce DIO. Third, since most publications use male mice, it is possible to compare ongoing studies to previously published work. A significant con when studying diseases specific to females is that females do not gain as much weight as males. To induce sufficient DIO, researchers must maintain mice on a high fat diet for extended periods, often 4–5 months. Some researchers remove the ovary to cause weight gain, but ovariectomy is used to generate a postmenopausal state, which may not be ideal for what the study design intends. Likewise, since Jackson Labs does not stock DIO females, responding to reviewers or implementing new therapies in DIO mice takes a long time. These long-term studies to induce DIO in female mice are costly. Furthermore, female DIO studies typically need larger sample sizes (*N*) to have sufficient statistical power due to lower weight gain compared to low fat diet control. Last, there are fewer established experimental paradigms in the literature to aid in study design or compare ongoing studies to existing literature. Yet including female DIO studies is imperative for reasons detailed below, where we focus on further studies regarding the VSG.

As of 2010, women were more likely to undergo bariatric surgery than men, as discussed above [201]. However, a model to study bariatric surgery on female rodents with DIO was yet to be published at that time. In 2013, Brinckerhoff et al. published that female Sprague Dawley rats fed a 60% high fat diet that underwent VSG causing weight loss, reducing leptin levels, and improving adiponectin concentrations compared to sham surgery controls [202]. Grayson et al. demonstrated that VSG improves many of the comorbidities of metabolic syndrome in female Long-Evans rats [203], just as had been previously reported in males. However, this group later showed that females and males respond differently to the surgery, with females showing changes in the regulation of lipid metabolism genes that are not observed in male rats [204]. Spann et al. also performed VSG on Long-Evans female rats fed a 40% high fat diet to examine the effects of bariatric surgery on the immune systems of mother and offspring [205, 206]. Mothers with VSG-induced reversal of DIO had lower levels of circulating T cells and increased placental permeability, ultimately leading to a high percentage of fetal demise [205]. Surviving offspring of female dams with previous VSG experienced early decreases in immune competency before rebounding later in life [206]. VSG on rats is easier simply due to the large size of the animal, but bariatric surgery on female mice has also been conducted. We have reported in C57BL/6 J female mice, that DIO induced significant weight gain, increased adiposity, and leptin compared to lean low fat fed controls, which was lost in DIO mice after VSG [207]. In our study, we aimed to mimic epidemiologic findings of reduced breast cancer risk after bariatric surgery in patients discussed above. We reported that DIO induced breast cancer progression using a syngeneic transplant model. Importantly, after weight loss by VSG, tumor progression was reduced. We showed that responsiveness to immune checkpoint blockade (ICB) immunotherapy was increased after VSG, but not in DIO mice, suggesting unique changes after bariatric surgery that primed for elevated efficacy of therapy [207]. In sum, despite limitations of extended time and costs using female mice to reach the extremes of obesity on a high fat diet compared to male mice, the use of female mice in DIO studies undergoing bariatric surgery is increasing. However, studies involving female models of bariatric surgery are still in the minority, comprising only 19% of the papers published on PubMed at the time of this review. With increasing interest from the National Cancer Institute (NCI) in benefits of bariatric surgery on cancer risk and outcomes, it is likely that increasing bariatric surgery studies on both male and female pre-clinical models are imminent.

## Mechanisms of improved metabolic outcomes associated with surgical weight loss

### Bariatric surgery and metabolic signaling

Obesity is a mediator of dysfunction in normal adipose tissue, mammary gland fat pads, or the breast [9, 208–212]. Of this dysfunction, there are three common mechanisms related to obesity-associated cancer risk: impaired insulin and metabolic signaling, altered sex hormone metabolism, and dysregulated inflammatory conditions [213]. Bariatric surgery and other weight loss methods may impact each of these mechanisms, though the mechanistic details are under active investigation [214]. It is currently unclear whether certain benefits of bariatric surgery are associated with the surgery itself or the resulting weight loss.

Increased insulin sensitivity was noted as a beneficial outcome of bariatric surgery as early in the development and characterization process as 1949 [215]. Further investigation has shown that changes to insulin sensitivity are merely a single component in the more notable changes to glucose metabolism correlated with bariatric surgery that is highly beneficial to reversing some of the more commonly obesity-associated comorbidities: type 2 diabetes mellitus, hyperlipidemia, and hypertension [118]. As intestinal hormones are stimulated following bariatric surgery to enhance insulin secretion, metabolic regulation is shifted to improve glucose metabolism [215]. Furthermore, increased insulin sensitivity is linked to shifts in immune phenotype, promoting anti-inflammatory aspects of immunity such as Th2 differentiation and systemic decrease in reactive oxygen species (ROS) [216]. These shifts in ROS production are significant in terms of cancer risk and promotion; ROS is a known inducer of DNA damage and genetic instability (discussed below). Likewise, insulin resistance has been connected to the development and prognosis of breast cancer [217–219]. Therefore, surgery-associated reductions in glucose and insulin, improvements in insulin sensitivity, and impacts on various pathways, including immune and oxidative stress, are mediators of the metabolic benefit associated with bariatric surgery. Recent reviews have reported on further potential mechanisms such as incretin hormone responses, bile acid and bile acid receptor signaling, and microbiota changes [119, 220, 221], not discussed in detail herein.

Leptin is an adipokine released by adipocytes involved in a range of cellular functions, from proliferation and angiogenesis to differentiation and inflammation [219, 222]. Increased serum levels have been associated with breast cancer occurrence and tumor aggression across all subtypes [222, 223], primarily in obese and/or postmenopausal women [224]. Leptin promotes tumor initiation, development, proliferation, and metastasis through various signaling mechanisms [225, 226]. Leptin also induces ROS production by activating NADPH oxidases and influences the production of pro-inflammatory cytokines [227]. Therefore, decreased leptin is an essential mediator of beneficial outcomes associated with bariatric surgery and reduced adiposity [184, 202].

Another obesity-associated factor impacted by bariatric surgery is estrogen signaling. Increased levels of white adipose tissue (WAT) are accompanied by increased aromatase activity [219]. Enhanced aromatase activity results in the rise of circulating sex hormones such as estradiol and other estrogens. As mentioned previously, weight loss by lifestyle intervention has been shown to reduce these circulating levels [77, 94], as does bariatric surgery-induced weight loss [228]. Lowered estrogen exposure reduces the risk of developing ER-positive breast cancer [225]. However, a concern after bariatric surgery is decreased bone density, which could also be attributed to reduced estrogen levels [229, 230]. Therefore, bariatric surgery-induced weight loss and reductions in adiposity would be beneficial in reducing overall estrogen concentrations, but this reduction comes with potential side effects that must be monitored. Interestingly, as noted above, the most significant impact on risk reduction after bariatric surgery was in ER-negative patients where estrogen signaling is not occurring in tumors due to low or absent ER expression. These complex interactions must be further studied to understand the benefits of bariatric surgery.

### Bariatric surgery and the immune system

Excess amounts of WAT have a remarkable impact on the immune system systemically and in tissues such as adipose depots [231–234]. Obesity impacts various immune cells, altering the proliferation of resident cells or recruiting them to adipose depots such as the epithelium surrounding the mammary gland or the mammary fat pad, resulting in an imbalance of pro-inflammatory and regulatory or anti-inflammatory mechanisms that can impair protective immunity to increase cancer risk and progression [231, 232, 235, 236]. As the extent of WAT increases and adipocytes expand with triglyceride storage, oxygen requirements exceed availability, leading adipocytes to become hypertrophic and undergo apoptosis [237]. These elevated levels of adipocyte expansion and cell death trigger innate immune responses and stimulate a pro-inflammatory immune state [238]. In the past two decades, the increase in our understanding of myeloid lineages, tissue-specific cell phenotypes, and innate and adaptive immune cell changes between preclinical models and humans has been immense [232]. The current understanding is that low-level, chronic (“smoldering”) inflammation resulting from obesity can cause oxidative stress, lipid peroxidation, and DNA damage as well as poor DNA repair, which results in genetic instability [67, 239, 240]; together, these factors can predispose cells to cancer initiation. Genetic instability (i.e., DNA integrity and stability) is necessary for tumorigenesis [208].

However, a sometimes-complicated notion is that obesity also leads to compensatory immunosuppressive mechanisms to avoid a constant, full-blown pro-inflammatory response, similar to controlling any immune reaction, to maintain homeostasis. These mechanisms include reduced absolute numbers of activated CD8 + T cells, T cell suppression by PD-1 [241, 242], T cell exhaustion [243], and dysfunctional NK cells [244]. Thus, obesity induces a complex and dynamic state of pro-inflammatory immune cell content and function with an influx of monocyte-derived “M1”-like macrophages, combined with reductions in immunomodulatory/regulatory or dysfunctional immune cells such as regulatory T cells (Tregs), “M2”-like macrophages, or NK Cells, together with an induction of checkpoint ligands (discussed below) [245–248], and elevated immunosuppressive cells such as immature monocytes or myeloid-derived suppressive cells (MDSCs) [249–253]. Peripheral Treg levels are reduced in correlation with increasing adiposity in humans [254, 255]. Fat resident Tregs, however, are a specific subtype of Treg cells that are abundant in adipose tissue [256], are increased in obesity [254], and likely perpetuate the obese phenotype. Paradigm shifting work described the importance of myeloid cells in adipose tissue which established adipose tissue and not merely a storage depot but an active immune depot. Macrophage infiltration to WAT was reported in back-to-back seminal publications in 2003 by Xu and Weisberg [257, 258]. Over the past two decades, a large body of work has shown that macrophages in obese adipose display a mixed phenotype that is dependent upon the extent and duration of obesity, specific adipose depot, site of origin of myeloid precursor, and severity of insulin resistance [259, 260]. Together, obesity’s chronic inflammation may increase the risk of immune-associated conditions such as cancer while suppressing levels of immunity that may protect against these conditions, or failed protective immunity.

Since obesity induces a pro-oncogenic state, it follows that weight loss may improve or reverse effects. Weight loss, specifically bariatric surgery, has been shown to reverse many of the impacts of obesity on the immune system, particularly low-grade inflammation and oxidative stress in both mouse models [261] and human studies [262–264]. Data on weight loss-induced changes to immunity after bariatric surgery is emerging. There is evidence that leukocyte infiltration of adipose tissue remains elevated above baseline for as long as 12 months following bariatric surgery in patients [265]. However, others have reported that surgically induced weight loss was shown to reverse this enrichment 3 months after gastric bypass in a human cohort [266]. Further, bariatric surgery decreased monocyte content and shifted circulatory T lymphocytes from Th2 to more pro-inflammatory Th1 [262, 267, 268]. Bariatric surgery has been indicated to restore pre-obesity ratios of M1/M2-like macrophages [269]. This polarization shift may be related to weight loss-induced decreases in the expression of molecules involved in macrophage chemotaxis and relief of the tissue hypoxia commonly found in obese patients [270]. Multiple studies have also described tissue-specific shifts in neutrophil populations, with increased neutrophils found in splenic and adipose tissue after bariatric surgery [265, 271–273]. Considerable work has been done to examine the effects of obesity, weight loss, and bariatric surgery on the B cell compartment to restore obesity-associated dysfunction [272–277]. With regard to cancer risk, studies must understand how bariatric surgery will impact specific immune subtypes, which depots (circulating, WAT, breast/mammary gland, or other tissues) will be affected, and how long these changes will persist after surgery. The advances in flow cytometry, reduced RNA sequencing costs, and increasing single-cell sequencing with advanced bioinformatics will transform our understanding of changes to immune cells with weight loss by bariatric surgery to help inform our understanding of cancer risk and impacts on cancer progression.

Another factor that impacts the immune system is the gut or extra-intestinal microbiome [119]. Bariatric surgery has been shown to increase the gut microbial richness and diversity, which is lost in obesity, though not increased to the levels observed in lean patients [278]. In this aspect, not all bariatric surgeries are created equal, with RYGB exhibiting more substantial alterations than gastric banding procedures [279]. Taking probiotics may help enhance the effects of bariatric surgery on microbiome richness. However, probiotics have only been examined in a few studies showing conflicting results between RYGB, where an improvement of microbiome diversity was reported following probiotic treatment, and VSG, which saw no such effects [280]. The increase in diversity may result from reduced comorbidities allowing for discontinuance of some medications [278] or the dietary and lifestyle changes encouraged in post-surgery patients [280, 281]. One study in a rat model observed that the changes in the microbiota occurred independently of weight loss following RYGB, indicating that other mechanisms beyond obesity and weight loss may be at work [282]. The impact of the gut and extra-intestinal microbes on cancer is a rapidly emerging field, mainly because gut microbes associate with and impact the response to immune checkpoint inhibitors [283–289]. Microbes and their microbially derived peptides and metabolites affect the enteric and systemic immune system, including the tumor microenvironment; the role of bariatric surgery, adaptive and innate immunity, and changes in the microbiome are areas of active study.

Overall, bariatric surgery may decrease the expression of pro-inflammatory cytokines and shift the phenotype of adaptive and innate immune cells towards the anti-inflammatory Th2 cells or M2-like phenotypes. This shift from a pro-inflammatory state to a regulatory state benefits tissue homeostasis. It reduces levels of cytokines and growth factors, improving comorbidities associated with obesity, such as insulin resistance. However, this loss of pro-inflammatory cells and increased regulatory cells is an apparent recipe for failed protective immune surveillance and increased cancer risk or progression through weak anti-tumor immunity. Yet, in long-term retrospective studies detailed above, epidemiologic evidence suggests the opposite: bariatric surgery reduces subsequent cancer risk. This implies that multiple factors associated with weight loss impact cancer risk. It is currently unclear as to whether the reversal of immune-mediated changes is entirely due to weight loss or instead due to the impacts of bariatric surgery, which are currently under active study. The investigation of alterations to metabolic pathways (i.e., reduced insulin, leptin, and estrogen) and varied immune cells, phenotypes, microbiome, and changes with time after bariatric surgery with weight loss are critical to examine.

## Bariatric surgery and immunotherapy

Obesity serves as a unique challenge for immunotherapy. Current strategies in immunotherapy target immunosuppressive proteins such as PD-1, PD-L1, and CTLA-4, among others. Immune checkpoint proteins are immune-inhibitory and depend on the crosstalk between the cancer cells, T cells, MDSCs, tumor-associated macrophages (TAMs), NK cells, and more [246, 290–294]. For the past decade, immune checkpoint blockade (ICB) has been a revolutionary intervention in some cancers such as melanoma, with just moderate effects in others, which is often due to the “cold” or immune excluded nature of some tumor microenvironments. Indeed, PD-1 and PD-L1 expression is correlated with worse outcomes in breast cancer patients [290, 295–302]. However, greater expression of these immune checkpoint proteins is also associated with improved responsiveness to immunotherapy. For example, combination therapy of nab-paclitaxel with ICB atezolizumab (anti-PD-L1 treatment) was efficacious for a minority of TNBC patients exhibiting high PD-L1 expression [303].

A limiting factor with ICB therapy is toxicity, including immune-related adverse events (irAEs) [66, 304]. Activation of the immune system by ICB in the obese state, which is already associated with inflammation, must be administered carefully to avoid exacerbating inflammatory mediators to inducing therapy-limiting complications [305]. In one study, a model of obese mice reported evidence of lethal cytokine storms in response to immunotherapy treatment. However, this study was not a cancer immunotherapy study [306]. Indeed, published studies in murine models have not demonstrated elevated lethality in obese mice [207, 307].

In fact, in obese patients, obesity has proven favorable in ICB treatment, as reviewed previously [65, 308]. PD-1 and PD-L1 expression or ligand-positive immune cells are increased by obesity, which likely allows for better efficacy of anti-PD-L1 treatments [245–247]. Obesity improves responsiveness to immunotherapy in melanoma and other cancers, but has not been reported for breast cancer [309–311]. One potential underlying mechanism is elevated inflammatory cytokines in obesity that stabilize PD-L1 or PD-1 [207, 248]. Thus, inflammation associated with the obese tumor microenvironment may synergistically increase PD-L1 or PD-1. We have begun to investigate the impact of VSG on breast cancer and immunotherapy. VSG itself induced PD-L1 which allowed for highly effective anti-PD-L1 immunotherapy in pre-clinical studies [207]. How obesity and weight loss, specifically by bariatric surgery, impacts the tumor microenvironment and systemic anti-tumor immunity, especially immune checkpoint ligands and immune cells, and response to ICB is under active investigation.

## Conclusions and future directions

**Fig. 3:**
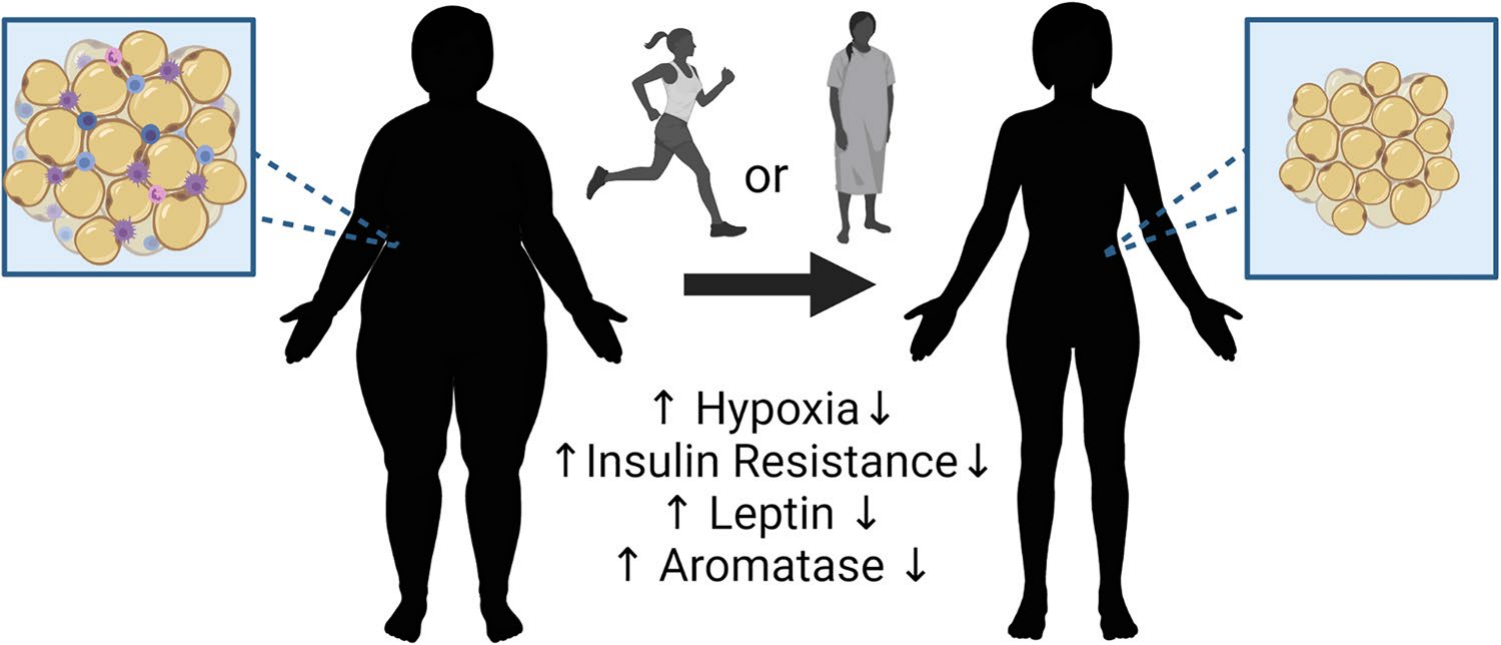
**Benefits of weight loss by lifestyle or surgical intervention**. The benefits associate with weight loss through lifestyle modifications or bariatric surgery include alterations in the mammary adipose with decreased hypoxia, insulin resistance, leptin release, and aromatase expression. These changes are associated with alterations in resident and non-resident immune cell populations that may influence the risk of onset and progression in the setting of breast cancer

With this review, we have highlighted the complexity of obesity’s impacts on risk and prognoses in epidemiological studies, preclinical models, and clinical trials. Obesity is associated with chronic low-level inflammation and hypoxia that can lead to genomic instability, elevated production of growth factors and adipokines such as estrogen and leptin, altered microbiota, and pro-inflammatory signaling combined with elevated immunosuppression. In general, obesity elevates cancer risk, which is highly consistent in postmenopausal breast cancer studies. Impacts on premenopausal breast cancer risk in different studies are varied from elevated risk of obesity to null to protective, likely depending on breast cancer subtype or methods to measure obesity. As specific subtypes and a more detailed evaluation of obesity metrics with the inclusion of menopausal status, race, and ethnicity are undertaken, we believe that the picture will become more evident. It is certain that obesity negatively impacts patient outcomes, recurrence, and survival in most settings. Weight loss before cancer onset or after treatment is beneficial. These benefits of weight loss are summarized in Fig. 3. While lifestyle interventions can reduce obesity and overweight, they often do not reach the critical levels necessary to induce positive changes and are difficult to maintain. Recent evidence suggests a long-term benefit of bariatric surgery that extends to greatly reduced cancer risks. Preclinical models, while sometimes variable, are beginning to disentangle the potential underlying mechanisms associated with dietary or bariatric surgery-associated weight loss. The impact of therapy, such as immunotherapy, on obesity-driven cancers and outcomes after lifestyle-associated weight loss or bariatric surgery is only beginning to be examined in patient populations and using preclinical models by our group and others. A deeper understanding of the immune cells, tissue signaling cascades, and cellular mechanisms underpinning these risks will lead to the identification of new targeted interventions aimed at improving outcomes. Future studies will ideally provide insight into biomarkers of obesity, immunosurveillance, and protective immunity for those at greater risk of cancer or point towards novel directions to improve therapeutic approaches in cancer patients.
